# Glucagon Can Increase Force of Contraction via Glucagon Receptors in the Isolated Human Atrium

**DOI:** 10.3390/ijms26020698

**Published:** 2025-01-15

**Authors:** Joachim Neumann, Franziska Schmidt, Britt Hofmann, Ulrich Gergs

**Affiliations:** 1 Institute for Pharmacology and Toxicology, Medical Faculty, Martin Luther University Halle-Wittenberg, 06097 Halle, Germany; franziska.schmidt.ue@gmail.com (F.S.); ulrich.gergs@medizin.uni-halle.de (U.G.); 2 Department of Cardiac Surgery, Mid-German Heart Center, University Hospital Halle, 06097 Halle, Germany; britt.hofmann@uk-halle.de

**Keywords:** glucagon, glucagon receptor, exenatide, human atrium

## Abstract

Glucagon can increase the force of contraction (FOC) in, for example, canine hearts. Currently, whether glucagon can also increase the FOC via cAMP-increasing receptors in the human atrium is controversial discussed. Glucagon alone did not (up to 1 µM) raise the FOC in human right atrial preparations (HAP). Only in the additional presence of the phosphodiesterase (PDE) 3 inhibitor cilostamide (1 µM) or 1 nM isoprenaline did glucagon raise the FOC, starting at 1 µM. The positive inotropic effects of glucagon in HAP were attenuated by a glucagon receptor antagonist (1 µM SC203972), but not by 100 nM exendin(9-39), a glucagon-like peptide-1 receptor (GLP-1R) antagonist. Glucagon (in the presence of cilostamide) demonstrated a reduced efficacy in elevating the FOC in HAP when compared with isoprenaline. In contrast to glucagon, exenatide alone, a GLP-1R agonist, starting at 1 nM, increased the FOC and was more potent and effective than glucagon in raising the FOC in HAP. The effects of exenatide on the FOC were attenuated by exendin(9-39). Hence, glucagon and GLP-1R agonists act functionally via different receptors in the human right atrium. Clinically, these data suggest that endogenous or exogenous glucagon can stimulate glucagon receptors in the human atrium, but only in the presence of PDE inhibitors.

## 1. Introduction

**Figure 1:**
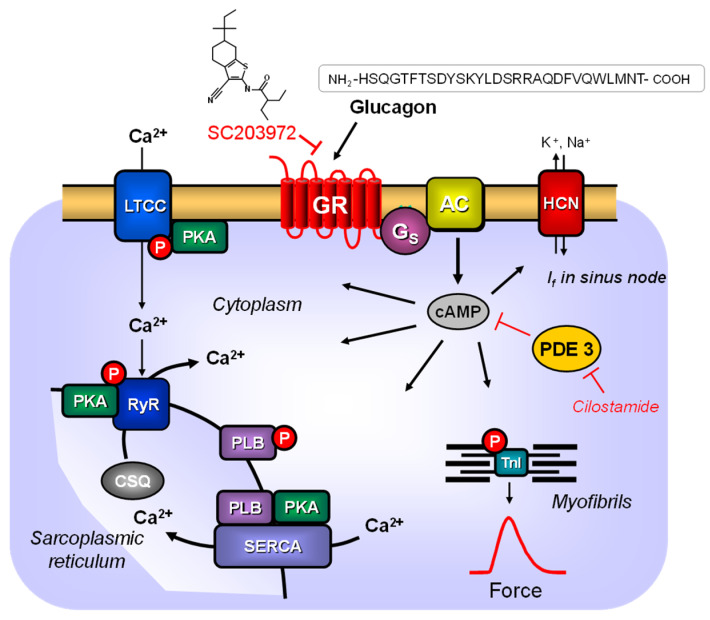
Putative mechanism(s) of action of glucagon in cardiomyocytes. Glucagon stimulates the glucagon receptor (GR). Then, via stimulatory GTP-binding proteins, the adenylyl cyclase (AC) catalyzes the formation of cAMP. This cAMP activates a cAMP-dependent protein kinase (PKA) and PKA activates by phosphorylation (P) cardiac regulatory proteins such as the L-type Ca^2+^ channel (LTCC), the ryanodine receptor (RyR), phospholamban (PLB) or the inhibitory subunit of troponin (TnI). The cAMP is degraded by phosphodiesterases (PDE) that can be inhibited in part by cilostamide. *I_f_*-currents are indicated by HCN, which is directly activated by cAMP. The sarcoendoplasmatic Ca^2+^-ATPase (SERCA) pumps Ca^2+^ into the sarcoplasmic reticulum, where the Ca^2+^ binds to calsequestrin (CSQ). The myofibrils are responsible for the generation of force, which is symbolized here by a single muscle contraction over time. SC203972 acts as a competitive antagonist at the glucagon receptor.

Glucagon, a peptide with a molecular mass of 3485 daltons, comprises 29 amino acids (Figure 1). Glucagon is predominantly produced in the α cells of the pancreas by cleavage of the 160-amino acid protein proglucagon [1,2]. Glucagon was one of the first drugs shown to increase the activity of adenylyl cyclases in the animal liver [3].

The primary physiological function of glucagon, via glucagon receptors, is the regulation of nutrient metabolism. In this context, over the past few decades, numerous studies on animals and humans have been conducted (overview in [4]). The antagonism of glucagon receptors has been demonstrated to elicit several physiological effects, including a reduction in blood glucose levels, a decrease in hepatic lipid oxidation, an increase in amino acid concentrations in the blood, the proliferation of α cells, the proliferation of β cells, and, of particular importance, cardioprotection [4]. Conversely, glucagon receptor activation has been shown to facilitate the catabolism of nutrients such as amino acids, lipids, and carbohydrates. Furthermore, glucagon receptor agonism has been shown to increase blood pressure and exacerbate ischemic heart disease [4].

Of relevance to the present study is the fact that glucagon can affect cardiac function, such as increasing contractility and/or beating rate, in living animals or in isolated animal hearts [5]. The positive inotropic effects and positive chronotropic effects of glucagon in the mammalian heart were initially explained by its action on β-adrenoceptors [5]. However, subsequent studies revealed that glucagon could enhance the force of contraction in canine hearts even in the presence of propranolol, a β-adrenoceptor antagonist [6,7]. This finding led to the hypothesis that a glucagon receptor might exist. Years later, the glucagon receptor was cloned [8]. Meanwhile, the structure of the human glucagon receptor has been elucidated [9]. The glucagon receptor belongs to the class B G-protein-coupled receptors, which differ from class A receptors (rhodopsin-like receptors, including beta-adrenoceptors) mainly due to the presence of a particularly large extracellular ligand-binding pocket, which is suitable for binding peptides such as glucagon [9]. Glucagon receptors were detected at the mRNA level, e.g., in the rat heart [8], and the highest expression of the glucagon receptor mRNA was detected in the sinoatrial node. Lower expressions were detected in the atrium, and no expression was noted in adult rat ventricular cardiomyocytes [10,11]. Accordingly, the mRNA of the glucagon receptor in adult mice showed higher expression levels in the right atrium than in the left atrium [12,13], whereas no mRNA expression by the glucagon receptor was detectable in the ventricles of adult mice [12,13].

Some studies have observed an elevation in heart rate and/or contractility in volunteers or patients who have been administered glucagon. However, other studies have not detected such cardiac effects of glucagon in humans (see overview in [14]). In isolated paced human ventricular preparations, some reports have noted that 1 µM glucagon and higher concentrations of glucagon increased the force of contraction [6,15]. However, subsequent studies failed to detect any inotropic effect of glucagon in either isolated human ventricular or atrial preparations [16].

In vitro glucagon stimulation of glucagon receptors in transfected cells results in an increase in cAMP levels, analogous to the stimulation of β-adrenoceptors by isoprenaline (Figure 1). The cAMP generated in the human heart is subsequently degraded by phosphodiesterases such as PDE3, which is inhibited by cilostamide. The glucagon-like peptide-1 receptor (GLP-1R) is believed to utilize the same signal transduction pathway, as evidenced by its stimulation by exenatide or semaglutide, which leads to positive inotropic effects in the human atrium [17]. Theoretically, this suggests that the positive inotropic effects of glucagon should be amplified by isoprenaline and cilostamide. Furthermore, it is hypothesized that the positive inotropic effects of glucagon are antagonized by drugs that are competitive antagonists for glucagon receptors, such as the small molecule SC203972 (Figure 1) (=compound **1** in [18]), but not by GLP-1R antagonists.

Thus, we tested the following hypotheses:

1. Glucagon exerts a positive inotropic effect in human right atrial preparations.
2. This positive inotropic effect of glucagon occurs via glucagon receptors in human right atrial preparations.
3. This positive inotropic effect of glucagon involves cAMP-dependent protein phosphorylation in human right atrial preparations.

Progress reports of this work were published in abstract form [19,20].

## 2. Results

**Figure 2:**
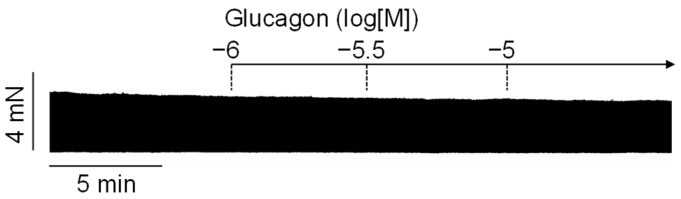
Original recording of the force of contraction of a human right atrial preparation. Glucagon alone, cumulatively applied at concentrations ranging from 1 to 10 micromoles per liter, did not exert any effects. The vertical bar indicates the developed force in millinewtons (mN), while the horizontal bar indicates the time in minutes (min).

In human right atrial preparations, glucagon alone was ineffective in increasing the force of contraction within 10 min of incubation at concentrations of 1 µM, 3 µM, and 10 µM (Figure 2), in accordance with prior reports by others [16].

**Figure 3:**
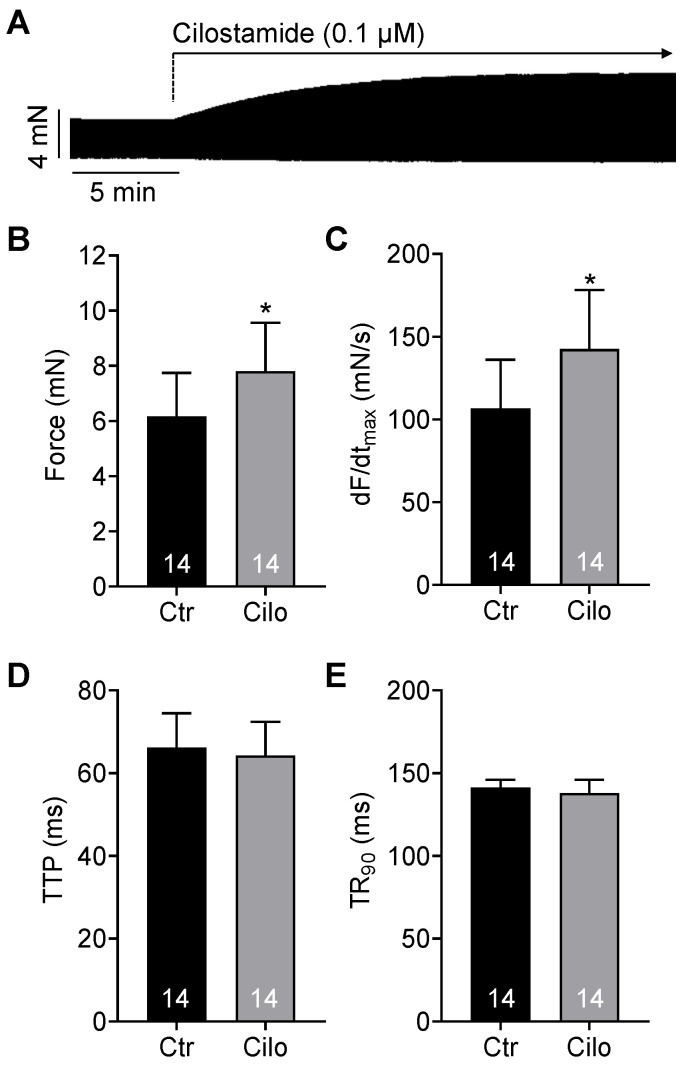
Positive inotropic effects of cilostamide on force of contraction. (**A**) Original recording of the force of contraction of a human right atrial preparation. Cilostamide alone induced a positive inotropic effect. The vertical bar indicates the developed force in millinewtons (mN), while the horizontal bar indicates the time in minutes (min). (**B**–**E**) Summarized effects of cilostamide alone on the force of contraction (**B**), the rate of tension development (dF/dt_max_, (**C**)), the time to peak tension (TTP, (**D**)) and the time to 90% relaxation (TR_90_, (**E**)). * *p* < 0.05 vs. control (Ctr = predrug value). The numbers in the bars indicate the number of experiments.

Therefore, we hypothesized that the effects of glucagon on the force of contraction in the human atrium might become measurable if the cAMP content in the heart was increased. This was achieved by using cilostamide or isoprenaline, followed by the subsequent application of glucagon without any washout steps. Our hypothesis thus turned out to be true. Cilostamide, a PDE3 inhibitor (Figure 1), increased the force of contraction per se. A typical recording for the effect of cilostamide is depicted in Figure 3A. Cilostamide increased not only the force of contraction (Figure 3B, *p* = 0.00009, effect size d = 1.488, test power (1 − β) = 0.9992), but also elevated the rate of tension development (Figure 3C, *p* = 0.00036, effect size d = 1.277, test power (1 − β) = 0.9927). Cilostamide did not shorten the time to peak tension and the time of relaxation (Figure 3D,E).

**Figure 4:**
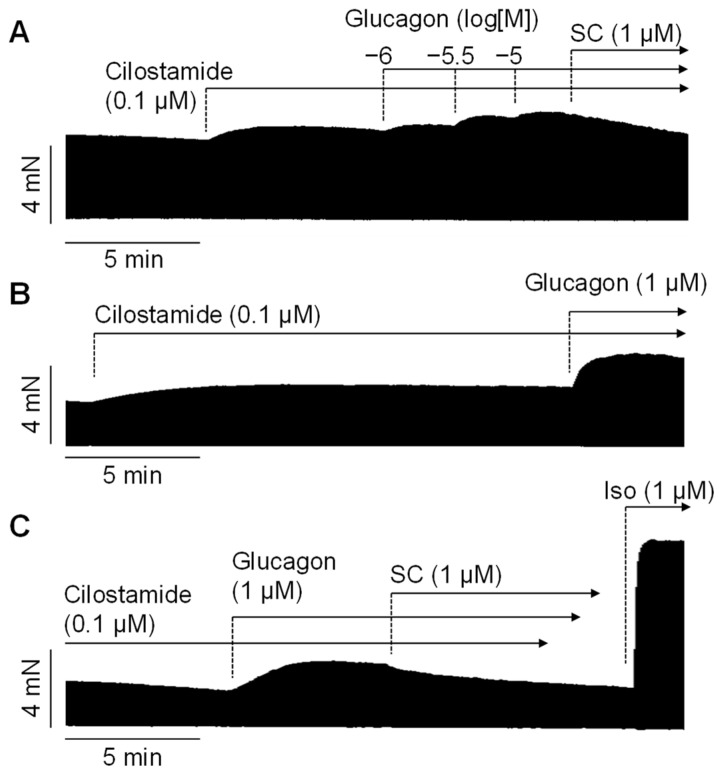
Original recordings of human right atrial preparations. In cilostamide pre-stimulated preparations, glucagon increased the force of contraction when cumulatively applied (**A**) or when, alternatively, only a discrete concentration of glucagon was applied (**B**,**C**). The glucagon receptor antagonist SC203972 (SC) diminished the effects of glucagon (**A**,**C**). The β-adrenoceptor agonist isoprenaline was much more efficacious in increasing the force of contraction compared to glucagon (**C**). The vertical bars indicate the developed force in millinewtons (mN), while the horizontal bars indicate the time in minutes (min).

**Figure 5:**
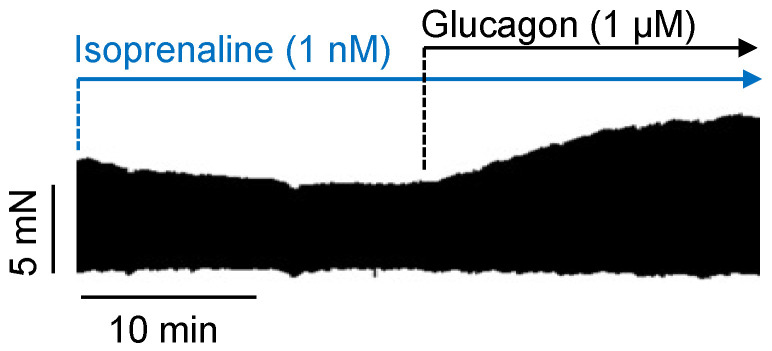
A discrete dose of glucagon exerted a positive inotropic effect after pre-stimulation of the human right atrial preparation by 1 nM isoprenaline. The vertical bar indicates the developed force in millinewtons (mN), while the horizontal bar indicates the time in minutes (min).

When cilostamide was first added to the organ bath to slightly elevate the force of contraction, subsequent additions of 1 µM, 3 µM, and 10 µM glucagon (in the presence of cilostamide) further increased the force of contraction. This is depicted in a typical original experiment in Figure 4A. In the presence of cilostamide, glucagon exerted a concentration- and time-dependent positive inotropic effect (Figure 4A). The same result was seen when glucagon was applied in a single high concentration of 1 µM (Figure 4B). Furthermore, the effect of glucagon in the presence of cilostamide could be reduced by the glucagon receptor antagonist SC203972 (Figure 4C). Glucagon (1 µM) was less effective than 1 µM isoprenaline in raising the force of contraction (Figure 4C). Moreover, in the presence of a low concentration of isoprenaline (1 nM), we noted a positive inotropic effect of 1 µM glucagon. This positive inotropic effect to glucagon in the presence of 1 nM isoprenaline amounted to an increase of 35 ± 12% (n = 4, *p* < 0.05). An original recording for this can be seen in Figure 5.

**Figure 6:**
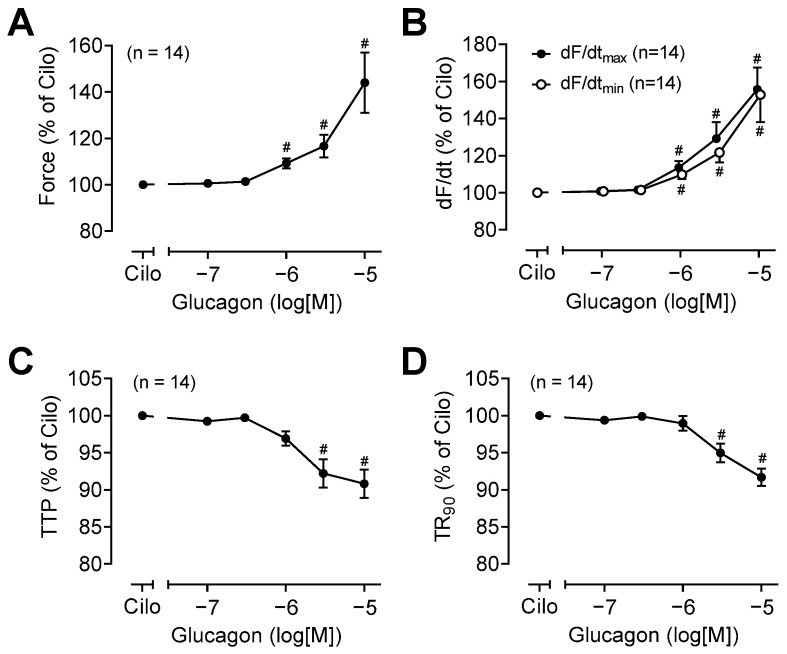
Summarized effects of cumulatively applied glucagon in presence of cilostamide on (**A**) the force of contraction (glucagon −6: *p* = 0.002, test power (1 − β) = 0.9895), (**B**) the rate of tension development (dF/dtmax) and the rate of tension relaxation (dF/dtmin) (glucagon −6: *p* = 0.003, test power (1 − β) = 0.867), (**C**) the time to peak tension (TTP) (glucagon −5.5: *p* = 0.007, test power (1 − β) = 0.952), and (**D**) the time to 90% relaxation (TR90) (glucagon −5.5: *p* = 0.007, test power (1 − β) = 0.943) in human right atrial preparations. # *p* < 0.05 vs. cilostamide (Cilo). The numbers in the brackets indicate the number of experiments.

The effect of glucagon in the presence of cilostamide on the force of contraction (in %), the rate of tension development, and the rate of relaxation are depicted in Figure 6A and Figure 6B, respectively. Moreover, similar to isoprenaline, glucagon shortened the time to peak tension and the time of relaxation (Figure 6C,D).

**Figure 7:**
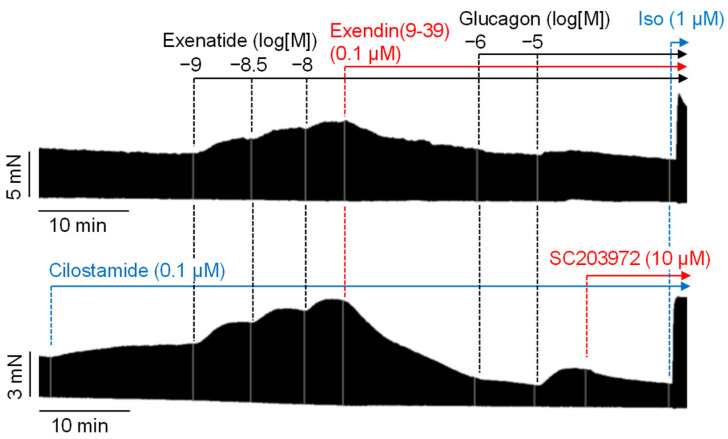
Stimulation of GLP-1 receptors and glucagon receptors in human right atrial preparations, with (**bottom**) and without (**top**) prior stimulation with cilostamide. As indicated, exenatide, exendin(9-39), glucagon, or SC203972 were added at the specified concentrations. At the end, a maximal concentration of isoprenaline (Iso) was added. All compounds were applied without washout steps. The vertical bars indicate the developed force in millinewtons (mN), while the horizontal bars indicate the time in minutes (min).

Next, we wanted to rule out that glucagon acted via GLP-1 receptors. Glucagon and GLP-1 are different proteolytic fragments from their common ancestor proglucagon and hence receptor cross reactivity can possibly occur in human atrial preparations. To that end, it was studied whether GLP-1R-mediated effects could be detected under our experimental conditions. By application of the GLP-1R agonist exenatide, the GLP-1R was functionally characterized in human atrial preparations under our experimental conditions. In contrast to glucagon, exenatide alone, in the absence of cilostamide, increased the force of contraction (Figure 7). This is different from glucagon that only acted in the presence of cAMP-increasing agents. Exenatide was much more potent than glucagon in raising the force of contraction, and the effects of exenatide started at 1 nM. This is depicted in the original recordings in Figure 7. Moreover, the effects of exenatide were, in all likelihood, mediated by the GLP-1R because they were blocked by exendin(9-39). Exendin(9-39) is a proteolytic fragment of the GLP-1R agonist exendin-4 [21] and acts as an GLP-1R antagonist (Figure 7). Exenatide (10 nM, the highest concentration tested) was less effective than 1 µM isoprenaline (Figure 7 top). However, under these conditions (absence of cilostamide), glucagon additionally applied at 1 µM was unable to augment the force of contraction (Figure 7 top). When cilostamide was given first to raise the force of contraction, exenatide (Figure 7 bottom) raised the force of contraction; this effect was also blocked by exendin(9-39). However, glucagon (that is now in the presence of cilostamide) increased the force of contraction, though exendin(9-39) was still in the organ bath (Figure 7 bottom). Accordingly, the glucagon receptor antagonist SC203972 reduced the force of contraction. Additional 1 µM isoprenaline increased the force of contraction substantially, indicating that the muscle was not damaged (Figure 7 bottom). This suggests to us that glucagon did not act via GLP-1 receptors but via glucagon receptors because the GLP-1R antagonist was still present when glucagon was applied.

**Figure 8:**
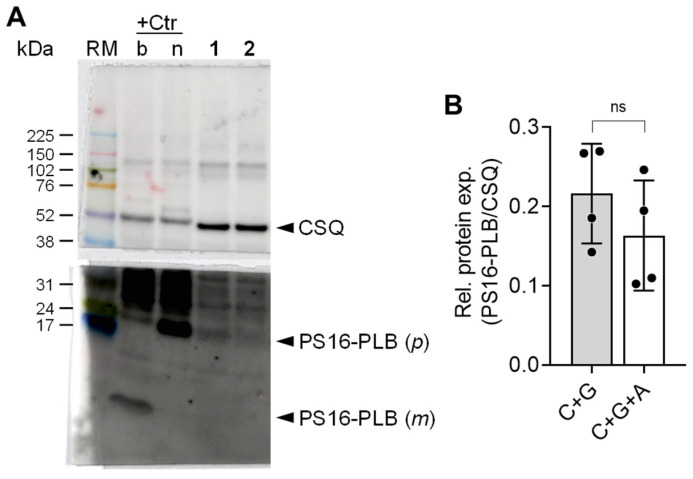
Glucagon-dependent phosphorylation of phospholamban (PLB). (**A**) Through Western blotting, the effect of 1 µM glucagon in presence of 0.1 µM cilostamide on PLB serine-16 phosphorylation (PS16-PLB) in human right atrial preparation could be demonstrated (sample 1). The effect was antagonized by additional application of 1 µM SC203972 (sample 2). PLB can usually be detected in its polymeric form (p) by Western blotting when loading non-heated samples. Boiling of the samples shifts the polymeric form to its monomeric form (m), which is not easily detectable. Here, we used an isoprenaline-treated mouse ventricular homogenate as positive control (+Ctr) that was loaded either non-heated (n) or after boiling (b) to demonstrate this shift and to identify the PLB band. The human samples were not heated. To ensure a comparable protein loading, the protein expression of calsequestrin (CSQ) was utilized as a cardiac myocytes-specific loading control. A pre-stained molecular weight marker (RM, rainbow marker) was used to identify the molecular weight range for cutting the membrane. (**B**) Quantification of the band intensities from four independent experiments, normalized to CSQ (PS16-PLB/CSQ). C, cilostamide; G, glucagon; A, antagonist: C+G corresponds to sample 1 and C+G+A corresponds to sample 2; ns, not significant.

Moreover, it can be asked whether the positive inotropic effect of glucagon was accompanied by a functionally relevant increase in cAMP-dependent protein phosphorylation. A well-established protein substrate for cAMP-dependent protein phosphorylation with functional relevance in the human heart is phospholamban. Accordingly, we studied the phosphorylation state of phospholamban by use of a phosphorylation-dependent antibody in a Western blotting experiment. This antibody detects the phosphorylated serine 16 in phospholamban, which is solely phosphorylated by the cAMP-dependent protein kinase. To this end, we freeze clamped the human atrial preparations in the presence of cilostamide and glucagon. As a control, samples were treated with cilostamide, glucagon and the glucagon receptor antagonist SC203972. The Western blot in Figure 8 demonstrates that glucagon increased the phosphorylation state of phospholamban at serine 16.

## 3. Discussion

### 3.1. Main New Findings

The main new finding of this study is that glucagon can exert positive inotropic effects in isolated electrically stimulated human atrial preparations via glucagon receptors, but only if the force has been pre-stimulated by an increase in cAMP content through the inhibition of, for example, phosphodiesterase 3.

### 3.2. Mechanism of Action of Glucagon

The effects of glucagon are likely mediated by cAMP for three primary reasons. Firstly, the positive inotropic effects occurred in the presence of cilostamide. Cilostamide, a PDE3 inhibitor, has been extensively utilized in human atrial preparations by our group and others. PDE3 is the predominant PDE in the human heart. Secondly, the positive inotropic effects of glucagon were visible in the presence of a small concentration of isoprenaline, a known stimulant of cAMP levels in the human heart. Thirdly, the positive inotropic effects of glucagon were accompanied by an increase in the phosphorylation state of phospholamban on serine 16, the cAMP-dependent phosphorylation site of phospholamban. Consequently, an elevation of the local compartment of cAMP has most likely occurred at least in the vicinity of phospholamban. This increased phosphorylation state can, at least in part, account for the increased rate of relaxation observed in the human atrial preparations.

The aforementioned effects are most likely glucagon-receptor-mediated. This conclusion is based firstly on the observation that the positive inotropic effects of glucagon were attenuated by the glucagon receptor antagonist SC203972. SC203972 has an IC_50_ value at human glucagon receptors of 171 nM [18]. Secondly, by default, we assume a glucagon-receptor-mediated effect because the positive inotropic effect of glucagon in the additional presence of cilostamide was not attenuated by exendin(9-39), a GLP-1R antagonist. The rationale behind exploring the potential involvement of the GLP-1R was as follows. While glucagon with the highest affinity binds to glucagon receptors, glucagon at 100-fold-higher concentrations can bind to GLP-1 receptors [22]. Hence, it seemed important to rule out any involvement of the GLP-1R. Exendin(9-39) blocks the binding of glucagon to GLP-1 receptors (the IC_50_ value at the human GLP-1R was 17 nM) but not the binding of glucagon to the glucagon receptors [22]. To confirm the efficacy of the utilized concentration of exendin(9-39) in our study, we conducted a series of experiments with exenatide, which had been reported by others to exert a positive inotropic effect in human atrial preparations and which had been shown to raise the phosphorylation state of phospholamban at serine 16 [23].

### 3.3. Comparison to Other Studies

Some groups have previously reported a positive inotropic effect of glucagon in isolated animal or human ventricular preparations (reviewed in [14]). As in our case, rather high concentrations of glucagon (1 µM or 5.7 µM or 10 µM) were required to increase the force of contraction (cat heart and dog heart: [6]; human left ventricular papillary muscle: increase by 16% in the rate of tension development [15,24,25]). This necessity for such high concentrations of glucagon is currently poorly understood. In contrast, in recombinant transfected cells, as little as 10 pM to 1 nM [6] glucagon is required to activate cAMP production by adenylyl cyclase [26,27]. On the one hand, the high requirement of 1 µM glucagon raises questions about the physiological role of glucagon in the heart, given that physiological concentrations of glucagon in the plasma typically range around 1 pM. On the other hand, one could argue that glucagon levels in the heart might be in the micromolar range because glucagon can be formed in the heart and might act in a paracrine or autocrine manner [14].

The observation that relatively high concentrations of glucagon are required to elicit force raised the suspicion that glucagon might not have acted by glucagon receptors but via GLP-1 receptors. Moreover, both receptors share similar signal transduction pathways via stimulation of cAMP-levels [28]. As the name implies, glucagon and glucagon-like-protein 1 share sequence homologies [1,28,29,30]. Thus, it is understandable why glucagon is also an agonist, but with affinities around 100 nM, at GLP-1 receptors [14]. Hence, we hypothesized that the positive inotropic effects of glucagon might really be mediated by high-affinity glucagon receptors rather than via low-affinity (for glucagon) GLP-1 receptors. Indeed, previous studies had reported that exenatide, an agonist at GLP-1 receptors alone, without pre-stimulation with cilostamide, could increase the force of contraction in the human heart [23]. These effects were found to be GLP-1R-mediated, as evidenced by the attenuation of the effects with exendin(9-39), a GLP-1R antagonist. It is noteworthy that both peptides are derived from a common precursor, proglucagon. We were able to reproduce and confirm their contractile data. Under our experimental conditions, exenatide was even more potent than in their study (1 nM versus 6 nM as first significant active concentrations of exenatide [23]). Nevertheless, even in the presence of exendin(9-39), glucagon raised the force of contraction, suggesting that glucagon does not act via GLP-1 receptors.

However, others failed to detect a positive inotropic effect of glucagon in the isolated human right atrium [16]. These data can be putatively reconciled with our present work; that means, glucagon alone, without previous stimulation of cAMP-levels, was indeed unable to raise the force of contraction. However, in the present study, glucagon was observed to enhance the force of contraction when cAMP production was enhanced (by isoprenaline) or cAMP degradation was impaired (by cilostamide). Similar experiments with augmented cAMP levels and subsequent application of glucagon were not reported by them [16]. Hence, we speculate here that a threshold concentration of cAMP is needed; once the threshold is reached, the human atrium is very sensitive to further, small increases in cAMP brought about by glucagon receptor stimulation.

### 3.4. Clinical Relevance

There are some accepted clinical indications of glucagon. Glucagon is mentioned in guidelines as a drug used to increase the beating rate in patients with low intrinsic beating rates. In certain cases, glucagon has been recommended as a treatment for intoxications involving β-blockers, such as propranolol, and calcium channel blockers, such as verapamil [31,32]. The beneficial effects of glucagon in these cases are believed to be attributable to its positive chronotropic effect. In light of the present work, glucagon’s direct positive inotropic effects may offer significant benefits in the management of such intoxications. However, it should be noted that glucagon exerts its effects only on the force of contraction and, by analogy, the beating rate, if cAMP levels in the heart are pre-stimulated. Indeed, such pre-stimulation might very well occur in the clinic. In severely ill patients, compensatory increases in β-adrenergic catecholamines are known to occur. Moreover, if patients with heart failure are given levosimendan or milrinone, which are PDE inhibitors, based on the present data, glucagon would be a positive inotropic agent. Furthermore, it is important that the cAMP hydrolyzing activity of PDE3, the main PDE in the human heart, can be inhibited by cGMP, which undergoes a significantly slower hydrolysis process [33]. Therefore, an elevation in cardiac cGMP levels, for instance, when natriuretic peptides are elevated under specific pathophysiological conditions, may also result in an increase in cAMP levels [34]. Furthermore, glucagon might enhance the effects of indirect sympathomimetics that are, for example, part of some over the counter cold medications or used as illicit drugs. In addition, under certain pathological conditions (e.g., atrial fibrillation, myocardial infarction, allergic reactions), the release of endogenous hormones such as serotonin or histamine from platelets or macrophages, respectively, would lead to a stimulation of cAMP levels in the heart. Hypothetically, glucagon has the potential to enhance the positive inotropic and chronotropic effects of serotonin or histamine in the human heart. These possibilities should be investigated in further studies.

### 3.5. Limitations of the Study

Methodological limitations: All experiments were performed with a fixed stimulation frequency of 60 beats per minute. However, it is known that the contractility of the heart is dependent on the heart rate. Thus, we cannot completely exclude the possibility that glucagon shows other effects, e.g., during tachycardia. However, the primary objective of the study was to demonstrate that glucagon exerts its effects on the human heart via glucagon receptors, and therefore the experiments were limited to one stimulation frequency. Nevertheless, the potential frequency dependency of glucagon effects may influence the clinical relevance of the findings.

It could be argued that the effects on the sinus nodes of humans have not been directly tested. Conducting such a study would necessitate access to the human pacemaker, which is not currently available. A further limitation of the study is the exclusion of data from the hearts of young adults, children, and neonates. It is conceivable that the function of glucagon in the human heart is age-dependent. An early study demonstrated age-dependent inotropic and chronotropic effects in sheep [35]. In rats, an age-dependent decline in glucagon-receptor-mediated signaling was noted [36]. In the mouse, a ventricular function in fetal hearts and neonatal hearts was reported (reviewed in [14]). However, due to the unavailability of samples from younger patients at our hospital, this particular investigation could not be undertaken. Likewise, the opportunity to study contractility in human ventricle tissue was precluded by a lack of access to that tissue. This is important because the expression of the glucagon receptor varies with age and across different regions of the heart, at least at the mRNA level [14]. Furthermore, an inotropic effect of glucagon in the ventricle would probably have more impact on overall cardiac function than in the right atrium. However, we are in the process of studying mice, wherein we overexpressed the human glucagon receptor specifically in cardiac myocytes. This model will facilitate the examination of the ventricular inotropic effects of human glucagon receptor stimulation.

Moreover, it should be considered that the atrial preparations were derived exclusively from ill patients, as healthy controls were not available. Therefore, the variability in patient comorbidities, medications, or age might affect the glucagon receptor expression, density, and/or signaling and, consequently, the contractility of the atrial preparations. Therefore, a generalization of the glucagon effects observed here is not readily feasible at this juncture.

## 4. Materials and Methods

### 4.1. Contractile Studies on Human Preparations

The contractile studies on human right atrial preparations were performed using the same setup and modified Tyrode’s solution as described previously [37]. The right atrial preparations were obtained during bypass surgery from 9 male patients and 4 female patients, aged 61–83 years. All patients included in the study suffered from coronary diseases (two- and three-vessel diseases). Cardiac drug therapy included apixaban, acetyl salicylic acid, furosemide, metoprolol and statins. In the laboratory, the samples were cut into small pieces that were mounted with metal hooks at each end of the muscle in a glass organ bath that was 10 mL in volume. The human atrial preparations were electrically stimulated (5-ms-long rectangular pulses; 60 beats per minute, bpm) with platinum electrodes with direct current from an Experimetria Exp-ST-CH4 stimulator (MDE, Budapest, Hungary). The voltage was set to 10 Volts to initiate contractions. By measurement of the preload tension, the muscles were stretched to the length where the maximum force was generated. The force of contraction was measured under isometric conditions after application of glucagon or other drugs to the organ bath (10 mL volume). The signals from the force transducer were electrically amplified and digitized using a PowerLab system (ADInstruments through their distributor in Oxford, UK) and finally stored on a commercial personal computer. The signals were quantified using commercial software (Lab Chart 8.0 from ADInstruments through their distributor in Oxford, UK). The methods used for atrial contraction studies in human samples have been previously published and were not altered in this study [38,39]. In some experiments, we finally applied antagonists and then isoprenaline as a positive control, as delineated in the figure legends.

### 4.2. Western Blotting

The homogenization of the samples, protein measurements, electrophoresis, primary and secondary antibody incubation, and quantification were performed following our previously published protocols [37,38]. In brief, the human atrial preparations were homogenized in a frozen state with SDS/bicarbonate solution to inhibit any enzymatic process. Per gel lane, 10 µg of these homogenates was loaded. We performed electrophoresis using pre-cast gels (Novex™ 4–20% “Tris-Glycine Plus Midi Protein Gels”, Invitrogen, Thermo Fisher Scientific, Waltham, MA, USA). Thereafter, proteins were electrically transferred to nitrocellulose membranes. Protein lanes were reversibly stained with Ponceau red and cut horizontally according to molecular weight. We incubated the upper parts of the nitrocellulose membranes with an anti-calsequestrin antibody (#ab3516, abcam, Cambridge, UK), and the remainder of the nitrocellulose membrane was incubated with an antibody against phospholamban phosphorylated at serine 16 (#A010-12 AP, Badrilla, Leeds, UK). Thereafter, secondary horseradish peroxidase-coupled antibodies were added, and signals were detected with an imager and quantified (Amersham ImageQuant 800, Cytiva, Freiburg im Breisgau, Germany). The ratio of serine 16-phosphorylated phospholamban and calsequestrin was calculated and is reported.

### 4.3. Data Analysis

The data shown are means ± standard error of the mean. Statistical significance was estimated using Student’s *t*-test or the analysis of variance followed by Bonferroni’s *t*-test as described in the legends. A *p*-value < 0.05 was considered to be significant. For statistical analysis and preparation of diagrams, the software Prism 9.0 (Graphpad Software, San Diego, CA, USA) was used. Effect sizes and the statistical power have been calculated using G*Power 3.1 [40,41].

### 4.4. Drugs and Materials

The isoprenaline used in this study was from Sigma Aldrich (Taufkirchen, Germany), the exendin(9-39) was from Th. Geyer (Renningen, Germany), and the exenatide from Biozol (Eching, Germany), SC203972 (N-(3-cyano-6-(1,1-dimethylpropyl)-4,5,6,7-tetrahydro-1-benzothien-2-yl)-2-ethylbutanamide; CAS 438618-32-7) was from Santa Cruz Biotechnology (Heidelberg, Germany) and glucagon from Bachem (Bubendorf, Switzerland). All other chemicals were of the highest purity grade commercially available. Deionized water was used throughout the experiments to prepare a modified Tyrode’s solution. Stock solutions were prepared fresh daily.

## 5. Conclusions

The hypotheses presented in the introduction can now be addressed as follows: only in the presence of cilostamide or isoprenaline is glucagon able to increase the force of contraction in human right atrial preparations. This likely occurs via glucagon receptors.

## Data Availability

The raw data supporting the conclusions of this article will be made available by the authors on request.
